# Analysis of Road Traffic Accident Fatalities in Karachi, Pakistan: An Autopsy-Based Study

**DOI:** 10.7759/cureus.14459

**Published:** 2021-04-13

**Authors:** Aiman Khurshid, Aruba Sohail, Maman Khurshid, Mir U Shah, Asra A Jaffry

**Affiliations:** 1 Forensic Medicine, Civil Hospital Karachi, Karachi, PAK; 2 Internal Medicine, Dow University of Health Sciences, Karachi, PAK

**Keywords:** autopsy, cause of death, pakistan, epidemiology, covid-19, fatalities, age factors, forensic medicine, road traffic accident, gender

## Abstract

Background

Road traffic accident (RTA) fatalities account for a significant number of unnatural deaths in Pakistan. Hence, it is necessary to investigate RTA fatalities in order to implement measures to reduce them. In the present study, we aimed to assess the detailed epidemiological characteristics of RTA fatalities by analyzing the data obtained from medico-legal autopsies performed at the Jinnah Postgraduate Medical Centre (JPMC) in 2019 and 2020. We assessed age- and gender-based variations in the pattern of RTA fatalities and determined the anatomical cause of death and sites of fractures among the fatalities. Moreover, we assessed the monthly distribution of cases in 2019 and 2020 to determine the impact of the coronavirus disease 2019 (COVID-19) on the number of RTA fatalities reported each month.

Methodology

In this retrospective study, data obtained from medico-legal autopsies of all RTA victims in 2019 and 2020 (n = 246) were collected from the Forensic Department of JPMC, Karachi. The data were then entered into Statistical Package for the Social Sciences version 24.0 (IBM Corp., Armonk, NY, USA) for analysis.

Results

The highest number of fatalities was recorded in the age group of 18-40 years (54.5%), while the lowest number was recorded in the age group of ≥60 years (8.5%). The male:female autopsy ratio was 6.03:1. Most fatalities were recorded from 6:00 am to 11:59 am (41.9%), followed by 12:00 pm to 5:59 pm (37.4%). Moreover, most victims (76.8%) died instantaneously within seconds to minutes of the incident. The number of RTA fatalities reported in 2019 (50.4%) was similar to that reported in 2020 (49.6%). However, the number of RTA fatalities reported in March-July 2020 was 35.6% lower than that reported in the same period in 2019, possibly because of the restrictions (such as lockdowns) that were imposed to control the spread of the COVID-19 pandemic in 2020. There was a statistically significant difference in the number of RTA fatalities reported in March-July 2020 and that reported in the remaining months of 2019 and 2020 (p = 0.006).

The cause of death was head injury in 159 (64.6%) cases and multiple traumatic injuries in 65 (26.4%) cases. Injury to the chest, abdomen, and pelvis caused death in 11 (4.5%), nine (3.7%), and two (0.8%) cases, respectively. Assessment of the site of fractures revealed skull fractures to be the most common type of fractures (53%), followed by rib/sternal fractures (19%). Upper limb and lower limb fractures occurred in 10% and 9% of the cases, respectively, while pelvic and neck fractures occurred in 6% and 3% of the cases, respectively.

Conclusions

Efforts need to be made at both government and individual levels to reduce RTA fatalities. Strict implementation of traffic laws is necessary. Although we noted a male preponderance, the reluctance to get females autopsied should not be disregarded. The significant decrease in RTA fatalities during March-July 2020 could be attributed to the reduced traffic burden due to the restrictions imposed to control the COVID-19 pandemic and the preventive measures taken, such as staying at home, to avoid contracting the virus.

## Introduction

According to the World Health Organization (WHO), approximately 1.35 million individuals die each year as a result of road traffic crashes and 93% of the world’s road traffic accident (RTA) fatalities occur in lower-middle-income countries, even though they have only 60% of the total vehicles in the world [1]. In a 2018 report, the WHO estimated that in Pakistan, RTAs result in 14.3 deaths per 100,000 population [2], which makes RTA fatalities an important public health issue that needs to be addressed immediately.

Worldwide, epidemiological studies have been conducted by extracting data from emergency departments of hospitals, websites, ambulance records, and police files [3-5]. However, medico-legal autopsies have been identified to be crucial for the epidemiological assessment of RTA cases. Studies conducted in Manipal [6] and Bengaluru [7] (India), Iran [8,9], Bangui [10], Australia [11], Mthatha and Transkei (South Africa) [12,13], Sri Lanka [14], and Kazakhstan [15] have used medico-legal autopsies for the assessment of RTA cases. Medico-legal autopsies help determine the exact cause and manner of death, time since death, and circumstances of death [16]. Analyzing these factors would help identify gaps in implementing preventive measures to curtail RTA fatalities. If such gaps are promptly addressed, RTA fatalities could be significantly reduced.

Age and gender are two of the most important risk factors to be considered when assessing RTAs. Of all age groups and genders, young males have been reported to be more prone to experiencing RTAs [9,17]. Hence, it is important to understand age- and gender-based variations in the pattern of RTA fatalities occurring in the populous city of Karachi in order to identify the population at risk and implement preventive measures. RTAs can also be classified according to the anatomical cause of death and site of fracture. Head and cerebral injuries have been found to be more prevalent in RTA fatalities [8-10]. Such information enables doctors in emergency departments to recognize the severity of RTA cases and provide prompt and accurate medical care accordingly.

The coronavirus disease 2019 (COVID-19) pandemic has significantly impacted all walks of life, and its devastating effects will resonate even in the years to come. The WHO declared COVID-19 as a pandemic on March 11, 2020 [18]. To curtail the spread of the virus, social distancing and lockdowns have been implemented worldwide. After the initial cases of COVID-19 were reported toward the end of February 2020, the government started imposing restrictions. Educational institutes were closed immediately. Moreover, businesses started instructing their employees to work from home. Crowded places, such as restaurants, were also closed. Although the aforementioned restrictions were imposed as soon as the initial cases were recognized, on March 21, 2020, the Government of Sindh formally announced a lockdown [19], significantly restricting movement. The lockdown was eased a bit on May 9 [20] to make amends to the economy; however, major restrictions, such as those on the transport sector, were lifted only on August 10 [21]. Hence, March-July 2020 could be considered the months when restrictions were imposed on movement to control the spread of the virus. During these months, transportation through all means was reduced like never before. Preventive measures, such as staying at home, were also taken by people so that they could protect themselves from contracting the virus. There was a decrease in RTA injuries and fatalities in Ireland [22] and Greece [23] because of the COVID-19 burden and the restrictions imposed. COVID-19 may have impacted RTA fatalities in Pakistan as well.

In the present study, we aimed to assess the detailed epidemiological characteristics of RTA fatalities by analyzing the data obtained from medico-legal autopsies performed at the Jinnah Postgraduate Medical Centre (JPMC), Karachi, in 2019 and 2020. We also assessed age- and gender-based variations in the pattern of RTA fatalities and determined the anatomical cause of death and sites of fractures among the fatalities. Moreover, we assessed the monthly distribution of cases in 2019 and 2020 to determine the impact of COVID-19 on the number of RTA fatalities reported each month.

## Materials and methods

This retrospective study was conducted at JPMC, one of the largest tertiary care hospitals in Pakistan. Data obtained from medico-legal autopsies of all RTA victims were collected from the Forensic Department of JPMC. The cases reported from January 1, 2019 to December 31, 2020 were retrieved and grouped according to the anatomical cause of death and site of fracture. The data were further stratified by the age group (1-<18, 18-40, 41-<60, or ≥60 years), gender (male or female), time of the day (12:00 am-5:59 am, 6:00 am-11:59 am, 12:00 pm-5:59 pm, or 6:00 pm-11:59 pm), and time between the incident and death (instantaneously, <1 h, 1-<2 h, 2-3 h, or >1 day). The data were entered into Statistical Package for the Social Sciences version 24.0 (SPSS, IBM Corp., Armonk, NY, USA) for analysis. The monthly distribution of the cases was separately analyzed for each year. Statistical significance was calculated using the t-test. All p-values <0.05 were considered significant. Graphs were generated at the end.

## Results

In total, 246 cases of RTA fatalities were autopsied at JPMC between January 1, 2019 and December 31, 2020. The highest number of fatalities was recorded in the age group of 18-40 years (n = 134, 54.5%), while the lowest number was recorded in the age group of ≥60 years (8.5%). The male:female autopsy ratio was 6.03:1 [211 (85.8%) males and 35 (14.2%) females]. Further subgroup analysis revealed that most RTA fatalities occurred in males aged 18-40 years (49.2%). Moreover, most fatalities were recorded from 6:00 am to 11:59 am (n = 103, 41.9%), followed by 12:00 pm to 5:59 pm (n = 92, 37.3%). Of the 246 cases reported, most victims (n = 189, 76.8%) died instantaneously within seconds to minutes of the incident (Table 1).

**Table 1:** Characteristics and pattern of RTA fatalities in 2019 and 2020. RTA, road traffic accident

Category	2019 (%)	2020 (%)	Total (%)
Total	124 (100)	122 (100)	246 (100)
Age (years)			
1–<18	15 (12.1)	14 (11.5)	29 (11.8)
18–40	66 (53.2)	68 (55.7)	134 (54.5)
41–<60	34 (27.4)	28 (23.0)	62 (25.2)
≥60	9 (7.3)	12 (9.8)	21 (8.5)
Gender			
Male	103 (83.1)	108 (88.5)	211 (85.8)
Female	21 (16.9)	14 (11.5)	35 (14.2)
Time of the day			
12:00 am–5:59 am	9 (7.3)	4 (3.3)	13 (5.3)
6:00 am–11:59 am	49 (39.5)	54 (44.3)	103 (41.9)
12:00 pm–5:59 pm	50 (40.3)	42 (34.4)	92 (37.4)
6:00 pm–11:59 pm	16 (12.9)	22 (18.0)	38 (15.4)
Time between the incident and death			
Instantaneously/within seconds to minutes	112 (90.3)	77 (63.1)	189 (76.8)
<1 h	9 (7.3)	43 (35.2)	52 (21.1)
1–<2 h	0 (0)	0 (0)	0 (0)
2–3 h	2 (1.6)	0 (0)	2 (0.8)
>1 day	1 (0.8)	2 (1.6)	3 (1.2)

**Figure 1:**
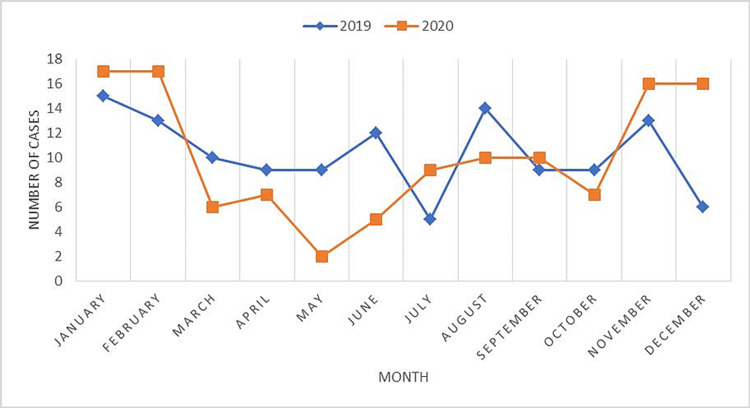
Monthly distribution of RTA fatalities. RTA, road traffic accident

When the yearly distribution of the cases was examined, the number of fatalities reported in 2019 (50.4%) was found to be similar to that reported in 2020 (49.6%) (Table 1). However, the number of fatalities reported during March-June 2020 was 35.6% lesser than that reported in March-June 2019 (Figure 1), possibly because significant restrictions, such as lockdowns, were in place to control the COVID-19 pandemic in March-July 2020. Moreover, there was a statistically significant difference (p = 0.006) in the number of fatalities reported in March-July 2020 and in that reported in the remaining months of 2019 and 2020 (Figure 1).

**Figure 2:**
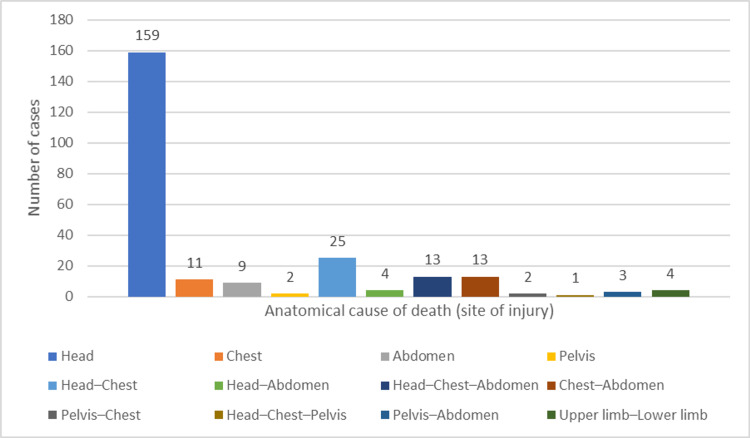
Distribution of RTA fatalities according to the anatomical cause of death. RTA, road traffic accident

The cause of death was head injury in 159 (64.6%) cases and multiple traumatic injuries in 65 (26.4%) cases. Injury to the chest, abdomen, and pelvis caused death in 11 (4.5%), nine (3.7%), and two (0.8%) cases, respectively (Figure 2).

**Figure 3:**
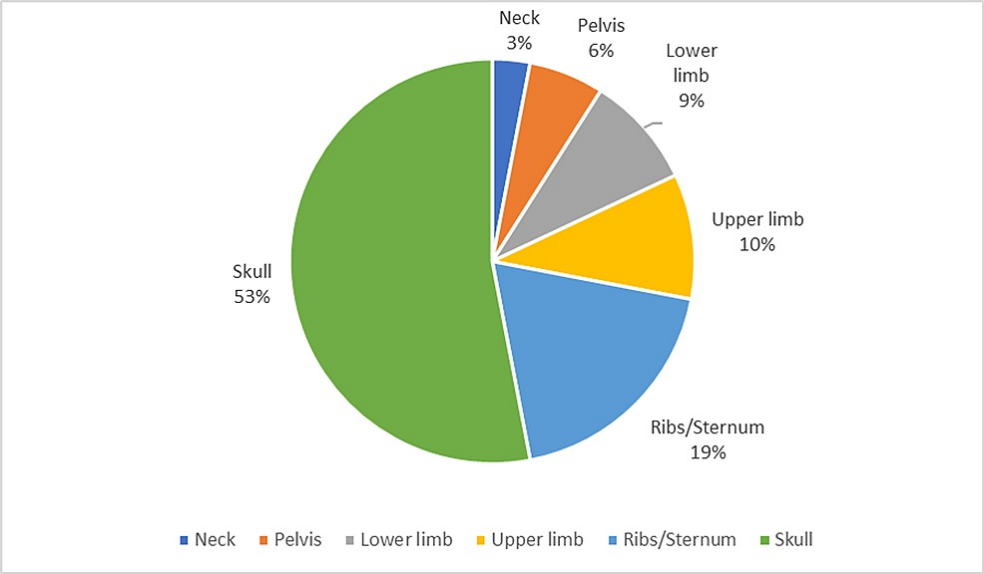
Distribution of RTA fatalities according to the site of fracture. RTA, road traffic accident

Assessment of the site of fractures revealed skull fractures to be the most common type of fractures (53%), followed by rib/sternal fractures (19%). Upper limb and lower limb fractures occurred in 10% and 9% of the cases, respectively, while pelvic and neck fractures occurred in 6% and 3% of the cases, respectively (Figure 3).

## Discussion

Medico-legal autopsies are performed as per the laws of each country, and the findings are admissible evidence in a court of law. Based on autopsy findings, a detailed epidemiological profile can be generated, which can help implement appropriate preventive measures in the future. RTA fatalities contribute to a significant proportion of medico-legal autopsies performed in Karachi, Pakistan [24]. This highlights the burden imposed by RTAs on the healthcare system in Karachi owing to the increasing number of vehicles, debilitating conditions of roads, and poor regard for safety measures.

Most RTA fatalities occurred in males aged 18-40 years (49.2%). These results are comparable with those conducted in Manipal (India) [6], Yazd (Iran) [8], Bangui [10], Mthatha (South Africa) [12], and Kazakhstan [15]. The gender and age variations noted in the present study are similar to those reported in previous studies conducted in Pakistan [24-26]. Males are more susceptible to RTAs because of cultural norms and a higher level of outdoor activities. Young males are also inclined toward risk-taking behaviors, such as speeding and driving their motorcycles on one wheel. These findings are very concerning as people of this age group are major contributors to the country’s economy and are generally the breadwinners in their families. Hence, the death of such economically productive individuals can prove to be a major loss.

The most common anatomical cause of death was head injury. In the present study, nearly three-fourth of the fatalities occurred as a result of head injury. The skull was found to be the most common site of fracture, thereby reinforcing this finding. Our findings are consistent with those of previous studies [6,8-10]. A previous study from Pakistan [24] also reported head injury to be the major cause of RTA fatalities. Hence, efforts should be made to increase the use of helmets among motorcyclists and seatbelts among car occupants. According to the Global Status Report on Road Safety 2018, although there is a national law on helmet and seatbelt use, only 10% of all riders wear helmets [2]. Law-enforcing agencies should take appropriate measures to ensure the implementation of these laws in order to curtail RTA fatalities. As 76.8% of the victims died instantaneously, immense efforts need to be taken to prevent RTAs. Increasing the number of pedestrian bridges and sidewalks, inspecting roads, and issuing fines for the violation of traffic laws, such as crossing the speed limit, are some preventive measures that could be imposed.

The Government of Pakistan imposed restrictions and lockdowns during March-July 2020 to control the COVID-19 pandemic. The number of RTA fatalities reported in these months was significantly lesser (p = 0.006) than that reported in the remaining months. An autopsy-based study in Greece also revealed a significant decrease in RTA fatalities during the lockdown period [23]. Similarly, a reduction in the number of RTA fatalities was observed in Peru during the lockdown period [27]. In addition to these restrictions, people took personal protective measures, such as staying at home and avoiding gatherings, because of the fear of contracting the virus. This attitude of people could also account for reduced traffic burden, resulting in a decrease in the number of RTAs. As 23.2% of the fatalities did not occur instantaneously, efforts need to be made to strengthen the emergency departments of hospitals so that a larger number of RTA victims can be saved. Prompt and effective medical care should be provided in this regard. Efforts should also be made to improve first aid services in the country.

The present study has some limitations. The retrospective method of reviewing medical records can potentially underestimate the true incidence of RTA fatalities. Although JPMC is one of the largest medical facilities in Karachi, the involvement of just one center resulted in a smaller sample size. This study could be repeated with a larger sample size, which would add weight to our conclusions. In many situations, RTA injuries and fatalities are not reported to the police because of beliefs that these incidents were destined to occur and were therefore unavoidable. Resistance to opt for autopsy is also observed, especially in the case of female victims, because of cultural and religious reasons. These reasons make it difficult to draw a clearer picture regarding the exact epidemiology of RTA fatalities.

## Conclusions

Efforts need to be made at both government and individual levels to reduce RTA fatalities. Interventions should particularly focus on young males as they account for a major proportion of RTA fatalities. Strict implementation of traffic laws should be ensured. Moreover, resources should be allocated for providing extensive driving education. Emergency departments should be well equipped to treat RTA victims effectively and promptly. In addition, funds should be allocated for improving ambulance and paramedic services so that victims can be taken to the nearest healthcare facility without delay. Although we noted a male preponderance, the reluctance to get females autopsied should not be disregarded. The significant decrease in RTA fatalities noted during March-July 2020 could be attributed to the reduced traffic burden due to the restrictions imposed to control the COVID-19 pandemic and the preventive measures taken, such as staying at home, to avoid contracting the virus.
